# Stable expression of H1C2 monoclonal antibody in NS0 and CHO cells using pFUSE and UCOE expression system

**DOI:** 10.1007/s10616-013-9615-x

**Published:** 2013-07-24

**Authors:** Suba Dharshanan, Heilly Chong, Swee Hung Cheah, Zulkeflie Zamrod

**Affiliations:** 1 Protein Science Department, Inno Biologics, Nilai, Negeri Sembilan Malaysia; 2 Department of Physiology, Faculty of Medicine, University of Malaya, Kuala Lumpur, Malaysia

**Keywords:** Humanized monoclonal antibody, Mammalian cell, pFUSE vector, Recombinant protein production, Ubiquitous chromatin opening element

## Abstract

From our recent publications, it was found that the deimmunization method (Dharshanan et al. (2012) Sci Res Essays 7:2288–2299) should be applied for the development of humanized anti-C2 monoclonal antibody (H1C2 mAb). However, the overlapping-PCR mutagenesis procedure used to insert the variable regions into cloning vectors was laborious and time-consuming. Additionally, the expression of H1C2 mAb in NS0 cells was low in static culture vessels. Therefore H1C2 mAb was redeveloped by deimmunization method with the following modifications in order to optimize the production of H1C2 mAb. First, instead of the overlapping-PCR mutagenesis procedure, synthetic DNA coding the variable regions were used to express the mAb. Second, two expression vectors, pFUSE and UCOE, were used to express H1C2 mAb in NS0 cells and CHO cells in order to investigate the combination that gave the highest number of high producing stable clones. This will provide the highest chance of finding clones with the requisite high productivity and stability required for manufacturing. We found that transfection of UCOE in CHO cells generated the highest number of high producing stable clones. To our knowledge, this is the first time that H1C2 mAb has been expressed in CHO cells.

## Introduction

In a recent publication (Dharshanan et al. 2012), we reported that the humanized anti-C2 monoclonal antibody (H1C2 mAb) developed using the deimmunization method in NS0 cells elicited the lowest immunogenic response in monkeys and therefore was most suitable for the development of humanized mAbs. In that paper we used the overlapping-PCR mutagenesis method to humanize potential immunogenic mouse residues. However, with mammalian cells this method is long and laborious as it frequently results in undesired mutations.

Mammalian cells, despite having lower yields, continue to be the system of choice because the other systems cannot adequately reproduce the post-translational modifications and glycosylation patterns that may be important for the tertiary structure of proteins (Browne and Al-Rubeai 2007). This is a vital factor because the lack of fidelity can lead to altered protein stability, lowered affinity to antigen, more rapid clearance rate and increased immunogenicity. These are major considerations especially if these mAbs are intended for repeated use as therapeutic agents in humans (Wurm 2004). Thus, Chinese hamster ovary (CHO) and NS0 cell lines have been most commonly used for the production of therapeutic antibodies (Zhu 2011). Nevertheless, the production of therapeutic proteins in mammalian cells is an expensive process with long development time (Browne and Al-Rubeai 2007). Hence, in order to increase culture yields and stability and at the same time decrease the duration for the development and production of humanized mAbs, it is crucial to optimize the in vitro mammalian cell production system.

In this publication, we have explored alternative methods from our previous paper in order to improve the chances of obtaining high-producing stable transfectomas that secrete H1C2 mAb more quickly and efficiently thus lowering the cost of production. First, the time-consuming and laborious overlapping-PCR mutagenesis procedure was replaced by the use of synthetic DNAs which were pre-designed to contain all desired humanized residues in its DNA sequences. Second, instead of the monocistronic pAH4602 and pAG4622 expression vectors that were employed previously, two different monocistronic expression vector systems, pFUSE and UCOE, as well as a bicistronic UCOE expression vector system were employed. The pFUSE vectors contain human antibody constant regions, while the UCOE expression vectors contain ubiquitous chromatin opening elements (UCOE) but lack the human antibody constant regions. Third, in addition to NS0 cells, CHO cells were also transfected with the pFUSE and UCOE expression vector systems. The results of the transfections with the different vectors and cell lines were compared and the combination that was most efficient in yielding the greatest number of stable transfectomas secreting high levels of mAbs was chosen for large-scale production.

## Materials and methods

In this publication, various forms of expression vectors containing synthetic genes were constructed from pFUSE and UCOE expression vectors (Dharshanan 2012). These vectors were transfected into NS0 and CHO cells as summarized in Table 1.

**Table 1:** Summary of combinations of vectors used for the expression of H1C2 mAb in NS0 and CHO cells

Cell line	Expression vector system used		
	pFUSE monocistronic vectors	UCOE monocistronic vectors	UCOE bicistronic vector
NS0	Linearized	Linearized	Linearized
	Unlinearized		
CHO	Linearized	Linearized	Linearized
	Unlinearized		

For pFUSE vectors both linearized and unlinearized vectors were employed whereas for UCOE vectors only linearized vectors were used

### Construction of expression vectors with synthetic genes coding the variable region of HIC2 mAb

#### Monocistronic pFUSE expression vectors

Monocistronic expression vectors, pFUSE-CHIG-hG1 and pFUSe2-CLIg-hk (Invivogen, San Diego, CA, USA) which contain the human IgG1 constant regions and the human kappa constant regions, respectively, were used to co-express the heavy and the light chains of H1C2 mAb. In previous experiments (Dharshanan et al. 2012) the physical DNA coding for the humanized variable regions were inserted into the expression vectors by the overlapping mutagenesis procedure; however, here the exact synthetic copies of DNA were designed and ligated into them instead. The physical DNAs, humanized-VH (hVH) and humanized-VL (hVL) (Dharshanan et al. 2011a) were substituted with the corresponding synthetic copies, syn-hVH and syn-hVL. The resulting recombinant vectors were named pFUSE-hVH (containing syn-hVH) and pFUSE-hVL (containing syn-hVL) (Dharshanan 2012).

#### Monocistronic UCOE expression vectors

Unlike monocistronic pFUSE vectors, the monocistronic UCOE vector (CET1019AS) (Millipore, Billerica, MA, USA) does not contain the human antibody constant regions. Therefore, in addition to the synthetic DNA coding the variable region of H1C2 mAb, synthetic DNA sequences coding the human antibody constant regions from pFUSE vectors were also designed and inserted. The synthetic genes designed were designated as follows:

1. syn-hVH-hCHm: this is the gene sequence coding the humanized heavy region of H1C2 mAb (hVH) and the human IgG1 constant region of heavy chain (hCHm)
2. syn-hVL-hCL: this is the synthetic gene sequence coding the humanized light region of H1C2 mAb (hVL) and the Ig Kappa constant region (hCL)

The resulting vectors were named pAS-hVL-hCL (containing syn-hVL-hCL) and pAS-hVH-hCHm (containing syn-hVH-hCH) (Dharshanan 2012).

#### Bicistronic UCOE expression vector

Like the monocistronic UCOE vector, the bicistronic UCOE vector (CET1019AD) **(**Millipore, USA) does not contain the human antibody constant regions. Therefore, the genes for both the light and heavy chains had to be inserted. Synthetic gene syn-hVL-hCL was used for the expression of the light chain. However, for the expression of the heavy chains, syn-hVH-hCHb which has *BstB*I and *Not*I restriction enzyme sequences at the 5′ and 3′ end, respectively, was used. A sequential cloning was performed for the ligation of both syn-hVL-hCL and syn-hVH-hCHb to CET1019AD and the resulting vector was named pAD-hVL-hCL-hVH-hCH.

### Transfection, selection and qualitative ELISA

The transfections of NS0 and CHO cells (both obtained from the ATCC, Manassas, VA, USA) were performed using a 6-well plate format and lipofectamine 2000 (cat. no.: 11668027, Life Technologies, Carlsbad, CA, USA). NS0 and CHO cells which had been adapted to serum-free media were used. NS0 cells were grown in hybridoma-SFM (cat. no.: 12045, Life Technologies, USA) containing 1 % (v/v) synthechol (cat. no.: S5442, Sigma-Aldrich, USA) and 1 % (v/v) glutamax (Life Technologies, USA) while CHO cells were grown in HyClone SFM4CHO medium (cat. no.: SH30518, Thermo Scientific, Logan, UT, USA), supplemented with 1 % (v/v) glutamax.

With monocistronic pFUSE vectors both NS0 and CHO cells were co-transfected with linearized pFUSE-VH and pFUSE-VL vectors as well as with unlinearized pFUSE-VH and pFUSE-VL vectors, since the pFUSE vectors are relatively small. However, with the monocistronic and bicistronic UCOE vectors, only the linearized forms were used because UCOE vectors are relatively large in size. The linearizations of pFUSE-VH and pFUSE-VL vectors were performed using *Not*I restriction enzyme, while pAS-hVL-hCL, pAS-hVH-hCHm and pAD-hVL-hCL-hVH-hCH vectors were linearized using *I*-*Sce*I homing endonuclease enzyme. For each well, a total of 4.0 μg of vector DNA were added. Thus for co-transfection, 2.0 μg of each monocistronic vector were used. Before transfection, each vector DNA was diluted in 250 μl of Opti-MEM reduced-serum medium (cat. no.: 51985034, Life Technologies, USA) using 1.5 ml centrifuge tubes. Using different 1.5 ml centrifuge tubes, 10 μl of lipofectamine were diluted into 250 μl of Opti-MEM reduced-serum medium and incubated at room temperature for 5 min. After that, the diluted vector DNA and lipofectamine were combined, gently mixed and incubated at room temperature for a further 20 min.

For transfection, approximately 10^6^ NS0 or CHO cells were pelleted by centrifugation at 1,000 rpm for 5 min and resuspended in 1 ml of Opti-MEM reduced-serum medium. The resuspended cells were added into each well of 6-well plates. Then, 500 μl of the lipofectamine-DNA complexes were added drop-wise to the cells in each well and the plates were then incubated in a 37 °C incubator with 5 % CO_2_ atmosphere for 4 h before the addition of 4 ml of the respective growth media for NS0 and CHO cells.

For controls (“mock transfections”) cells were similarly treated except that no DNA was added to the Opti-MEM reduced-serum medium. To remove non-transfected cells, selective media containing specific antibiotics were added 72 h post-transfection. For cells transfected with pFUSE vectors, 200 μg/ml of zeocin (Life Technologies, USA) and 2 μg/ml of blasticidin (Life Technologies, USA) were added while for cells transfected with UCOE vectors, 5 μg/ml of puromycin (Life Technologies, USA) were added. The antibiotic selections were performed until all mock transfected NS0 and CHO cells were non-viable. The viable parental transfectomas were then evaluated for H1C2 mAb productivity using qualitative ELISA as described (Dharshanan et al. 2011a). All transfections were done in triplicate.

### Isolation of high producer transfectomas using ClonePix FL system and characterization of H1C2 mAb

The screening and selection of NS0 and CHO transfectomas secreting high levels of H1C2 mAb were done as described in an earlier publication with minor modifications (Dharshanan 2012). In order to grow clones from individual separate transfected cells, 50,000 parental transfectoma cells secreting H1C2 mAb were added to 100 ml of semi-solid growth media. For NS0 transfectomas, the semi-solid growth media consisted of 90 ml semi-solid medium for hybridomas/myelomas (cat. no.: K8600, Molecular Devices, USA), 1 ml of glutamax, 1 ml of anti-human capture antibody conjugated to fluorescein isothiocyanate (FITC) (cat. no.: K8200, Molecular Devices, Sunnyvale, CA, USA) and 8 ml of sterile water. For CHO cells, the semi-solid growth medium for CHO cells (cat. no.: 8712, Molecular Devices, USA) was used instead. The cells and medium were mixed vigorously and 2 ml of each mixture were transferred to each well of 6-well plates (cat. no.: 3516, Sigma Aldrich, USA). The plates were then incubated at 37 °C, 5 % CO_2_ with high humidity for 14 days.

The transfectomas in semi-solid medium were then analyzed using the ClonePix FL system and high producer clones were identified and isolated as described (Dharshanan et al. 2011a). The isolated clones were scaled up in static cell culture vessels and at passage 10, qualitative and quantitative ELISAs were performed to evaluate the H1C2 mAb productivity. The functionality of the H1C2 mAbs secreted were characterized using SW1116 cells, a colorectal carcinoma cell-line expressing C2-antigen, as described previously (Dharshanan et al. 2011b).

## Results

### Construction of expression vectors

Various synthetic genes were inserted into pFUSE and UCOE vectors which were then transfected into cells for the expression of H1C2 mAb. A summary of the recombinant vectors and their theoretical sizes and actual sizes as determined by gel electrophoresis is shown in Table 2.

**Table 2:** Summary of the properties of the recombinant vectors used for the expression of H1C2 mAb in NS0 and CHO cells

Expression vector used	Recombinant expression vector	Contents	Theoretical size (kb)	Experimental size (kb)
Monocistronic pFUSE				
pFUSE-CHIg-hG1	pFUSE-hVH	syn-hVH (~0.5 kb) + pFUSE-CHIg-hG1 (~4.5 kb)	~5.0	~5.0
pFUSe2-CLIg-hk	pFUSE-hVL	syn-hVL (~0.4) + pFUSe2-CLIg-hk (~3.8)	~4.2	~4.2
Monocistronic UCOE				
CET1019AS	pAS-hVH-hCHm	syn-hVH-hCHm (~1.5 kb) + CET1019AS (~8.1 kb)	~9.6	~9.6
CET1019AS	pAS-hVL-hCL	syn-hVL-hCL (~0.7 kb) + CET1019AS (~8.1 kb)	~8.8	~8.8
Bicistronic UCOE				
CET1019AD	pAD-hVL-hCL- hVH-hCH	syn-hVL-hCL (~0.7 kb) + syn-hVH-hCHb (~1.5 kb) + CET1019AD (~11.0 kb)	~13.2	~13.2

For UCOE vectors both monocistronic and bicistronic vectors were employed whereas for pFUSE vectors only monocistronic vectors were used

### Transfection and expression of H1C2 mAbs

The viability rates of NS0 and CHO cells after transfection with various vectors are shown in Table 3. When NS0 cells were transfected with linearized pFUSE vectors, all 3 groups contained viable cells (resistant to the antibiotic used in the selective media). However, with unlinearized pFUSE vectors only 1 of the 3 groups of NS0 cells transfected contained viable cells. On the other hand, all three CHO populations transfected with linearized monocistronic vector and bicistronic UCOE vector contained viable cells after antibiotic selection. However, no viable cells were obtained for NS0 cells transfected with UCOE vectors and CHO cells transfected with pFUSE vectors.

**Table 3:** Viability of stable parental transfectomas after antibiotic selection

Expression vector system	Number of viable transfectomas (out of 3 transfections)	
	NS0	CHO
Linearized pFUSE-M	3	0
Unlinearized pFUSE-M	1	0
Linearized pUCOE-M	0	3
Linearized pUCOE-B	0	3

For the selection of stable transfectomas, zeocin and blasticidin were used for cells transfected with pFUSE vectors, while puromycin was used for cells transfected with UCOE vectors. All transfections were performed in triplicate. M denotes monocistronic vector while B denotes bicistronic vector

### Productivity of H1C2 mAb in NS0 cells using pFUSE expression vectors

**Fig. 1:**
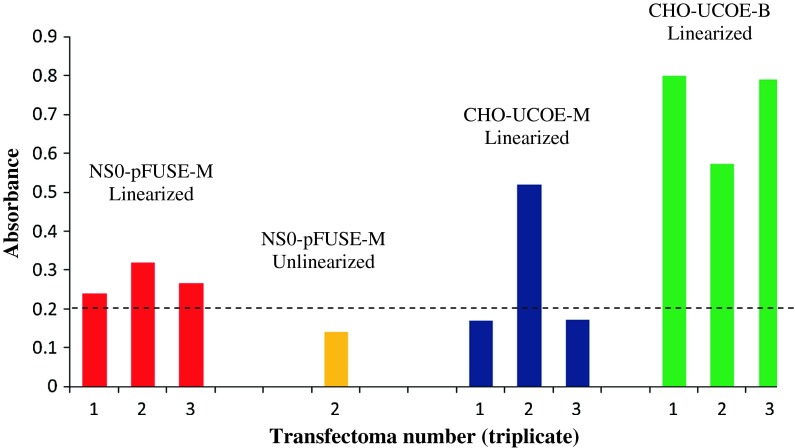
Qualitative ELISA of antibiotic resistant parental transfectomas. All viable transfectomas were analyzed for H1C2 productivity. All transfections in triplicate had 3 viable clones each except for the parental NS0 cell transfected with unlinearized pFUSE vectors, which had only 1 (transfectoma 2) viable parental clone. Cells were considered low-producing if the absorbance value was below 0.2 units. Although all CHO-UCOE-M were viable, two of the transfectomas (transfectoma 1 and 3) were low producers

The viable parental NS0 transfectomas generated with pFUSE vectors were then analyzed for H1C2 mAb productivity. As shown in Fig. 1, all three parental NS0 transfectomas transfected with linearized pFUSE vector (NS0-pFUSE-M) produced H1C2 mAb. However, the one viable NS0 transfectoma population (transfectoma-2) that was transfected with unlinearized pFUSE vectors failed to produce or produced at low levels despite the fact that the cells were resistant to the antibiotics used for the selection of the heavy and light chain of H1C2 mAb.

### Productivity of H1C2 mAb in CHO cells using UCOE expression vectors

The viable parental CHO transfectomas transfected with UCOE vectors were also analyzed for H1C2 mAb productivity. From Fig. 1, all the viable cells of the triplicate transfections with linearized bicistronic (linearized CHO-UCOE-B) produced substantial quantities of H1C2 mAb. However, only one of the three transfections with monocistronic UCOE vectors (CHO-UCOE-M) produced cells that secreted significant levels of mAbs, while the other two populations showed low productivities.

### Selection of high H1C2 mAb producing NS0 and CHO transfectomas using ClonePix FL system

**Fig. 2:**
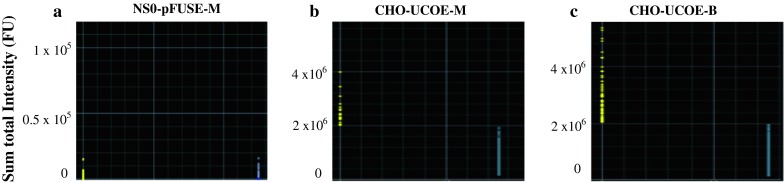
Dot-blot analysis of transfectomas using ClonePix FL system: Sum of total intensity (FU). From the fluorescence data whereby each colored dot represents one monoclonal transfectoma, NS0 cells transfected with linearized pFUSE vector **a** had lower fluorescence intensity compared to CHO cells transfected with either monocistronic **b** and bicistronic **c** UCOE vectors. *Yellow dots* and *blue dots* represent the isolated and non-isolated transfectomas, respectively. (Color figure online)

All producing transfectoma populations were then screened using the ClonePix FL system and ranked according to their sum total fluorescence intensities. Since the detection antibody (conjugated to FITC) used was specific to the human Fc region of human antibody, the total sum of the fluorescence intensity of each transfectoma would be a measure of the production rate of the whole H1C2 mAb. From the dot-blot data (Fig. 2), it was found that all parental NS0-pFUSE-M transfectomas (Fig. 2a) were low producers as their fluorescence intensities were less than 50,000 FU. The sum of total intensity of the highest NS0-pFUSE-M producer was only 19,898 FU.

On the other hand, more than 90 % of both CHO-UCOE-M (Fig. 2b) and CHO-UCOE-B transfectomas (Fig. 2c) had intensities of greater than 50,000 FU, indicating a higher level of mAb production rate. The sum total intensities of the highest producer of CHO-UCOE-M (Fig. 2b) and CHO-UCOE-B (Fig. 2c) were 3,985,878 and 5,357,028 FU, respectively. It was also found that CHO-UCOE-B (Fig. 2c) had a higher number of transfectomas with intensities of between 2 × 10^6^ and 4 × 10^6^ FU compared to that of CHO-UCOE-M (Fig. 2b). In fact, CHO-UCOE-B had 6 clones with a sum of total intensities greater than 4 × 10^6^ FU.

A total of 90, 27 and 122 monoclonal transfectomas of NS0-pFUSE-M, CHO-UCOE-M and CHO-UCOE-B, respectively, were isolated, scaled-up and evaluated for H1C2 productivity using quantitative ELISA at passage 10. Clones were categorized based on their productivity: (1) non-producers if the productivity was less than 1.0 μg/ml (2) low producers if productivity was between 1.0–10.0 μg/ml (3) average producers if it was between 10.0–50.0 μg/ml and (4) high producers if it was above 50.0 μg/ml.

**Fig. 3:**
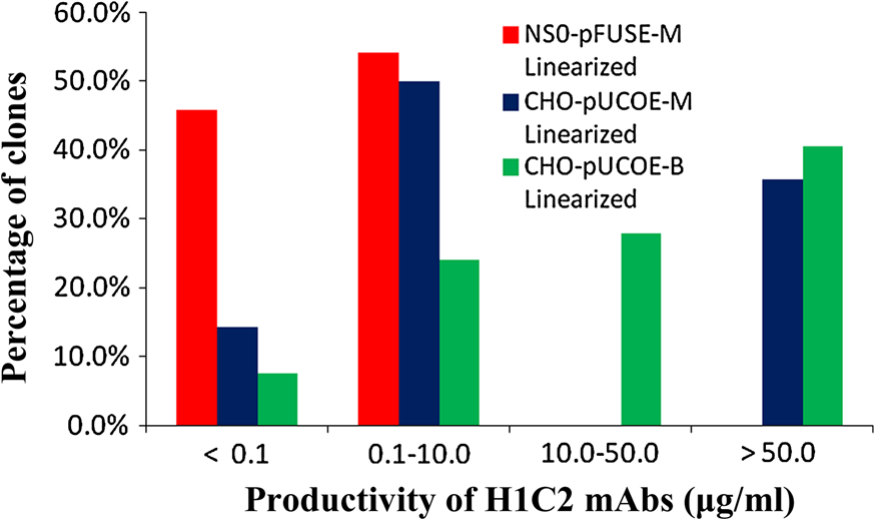
Quantitative ELISA of monoclonal high-producer transfectomas isolated using ClonePix FL system. A total of 90, 27 and 122 of the high-producing clones from each transfectoma were isolated, scaled-up and after 10 passages, were evaluated for their stability and productivity. By this passage approximately 45.8 % of the NS0-pFUSE-M were no longer producing H1C2 mAbs (<0.1 μg/ml) and the remaining clones were low producers (0.1–10.0 μg/ml). For CHO-UCOE-M, 14.3 % were non-producers, 50.0 % low producers and 35.7 % high producers (>50.0 μg/ml). For CHO-UCOE-B, 7.5 % were non-producers, 24.1 % low producers, 27.9 % moderate producers (10.0–50.0 μg/ml) and 40.5 % high producers

At passage 10, almost half of the NS0-pFUSE-M clones were non-producers and the remaining NS0-pFUSE-M (54.2 %) clones were all low producers (Fig. 3). In contrast, for the CHO-UCOE-M clones, 35.7 % were high producers, 50.0 % were low producers and 14.3 % were non-producers, while for the CHO-UCOE-B clones the numbers were 40.5 % high producers, 27.9 % moderate producers, 24.1 % low producers and 7.5 % non-producers.

Quantitative ELISA performed also showed that the highest productivity of NS0-pFUSE-M clones was approximately 0.6 μg/ml, while the productivity of high producers of CHO-UCOE-M and CHO-UCOE-B clones were between 50–110 μg/ml. No significant difference in H1C2 mAb productivities were found between CHO-UCOE-M and CHO-UCOE-B transfectomas, but productivities of both CHO-UCOE-M and CHO-UCOE-B transfectomas were at least 100 times higher than that of NS0-pFUSE-M transfectomas. A cell-based ELISA (Dharshanan et al. 2011b) also confirmed that H1C2 mAbs secreted by NS0-pFUSE-M, CHO-UCOE-M and CHO-UCOE-B were all able to bind to the C2-antigen expressed on the surface of colorectal cancer cells with similar affinity to our previously developed H1C2 mAbs.

## Discussion

In a previous publication (Dharshanan et al. 2012) we have compared the functionality and immunogenicity of humanized mAbs prepared from mouse IgG against the C2 antigen using a deimmunization method and a logical substitution method. We came to the conclusion that the deimmunization method was superior as the resulting mAbs had lower immunogenicity when tested in monkeys.

In those experiments, the humanized mAbs were prepared by site-specific overlapping mutagenesis in order to perform specific conversion of mouse residues to the corresponding human residues. However, this procedure is time consuming and relatively inefficient. Therefore we decided to employ the direct substitution of synthetically constructed DNA sequences with the corresponding human residues instead. We inserted the synthetic DNA into two different expression systems (pFUSE & UCOE) which were transfected into NS0 and CHO cells, and transfectoma viability and productivity of H1C2 mAb were compared.

From our data we found that the use of UCOE vector in CHO cells were consistent in generating high mAb-producing stable cells. The other combinations, pFUSE in NS0 cells, pFUSE in CHO cells and UCOE in NS0 cells either did not produce viable cells or had lower efficacy in producing cells that secreted high levels of mAb. We also concluded that for production of humanized anti-C2 mAb the use of bicistronic UCOE vector to transfect CHO cells had the greatest efficacy in generating large numbers of high producing transfectomas.

The transfection of NS0 cells with linearized pFUSE vectors resulted in all parental cell lines being viable after antibiotic selection, whereas with unlinearized vectors only one viable parental cell line was obtained. This could be due to the possibility that, after entering the cell, the unlinearized vectors might be cleaved non-specifically at random sites which could cause the antibiotic resistance gene or the antibody gene of interest to be destroyed, thus affecting cell viability and antibody production. On the other hand, linearization of the vector DNA before transfection by a single digestion with a selected restriction enzyme in a non-coding area of the gene may have the advantage of ensuring the integrity of all necessary gene elements of the vector, hence improving the survival and productivity of the transfectomas in selective medium (Stuchbury and Münch 2010).

The transfection of pFUSE vector into NS0 cells, even when successful, generally resulted in lower numbers of high producers than transfection of UCOE vector in CHO cells. This could be due to the possibility that the transgenes were inserted into regions that suppressed their expression. The integration of a transgene into, or close to, heterochromatin will result in it being silenced, whereas integration into actively transcribed euchromatin usually leads to transgene expression (Kwaks and Otte 2006). Because a large proportion of the genome is in the form of heterochromatin and insertion is random, the chance of a transgene integrating into, or close to, heterochromatin is high and the chance that the gene is silenced is also high.

Successful transfection of UCOE vectors into CHO was much more effective in generating high producer cells. One reason could be due to the fact that UCOE vectors contain ubiquitous chromatin opening elements. Ubiquitous chromatin opening elements are elements derived from the promoters of housekeeping genes which are usually transcriptionally active owing to a significant extent of histone acetylation. Therefore, employment of UCOE elements-incorporated expression vectors was reported to give major improvements in gene expression in stably-transfected mammalian cells. This is thought to be through UCOE’s effects on the structure of chromatin, preventing transgene silencing and giving consistent, stable and high-level gene expression irrespective of the chromosomal integration site (Benton et al. 2002). This is shown to be the case in the UCOE transfected CHO cells in this study which has been shown to generate higher numbers of high producer cells secreting the humanized anti-C2 mAb compared to pFUSE vectors.

However, there is a difference in the effectiveness of monocistronic and bicistronic UCOE vectors in producing CHO transfectomas that secrete H1C2 mAb. Transfection of CHO cells with monocistronic UCOE vectors and selection with antibiotic resulted in all parental transfectomas being viable, but only one population produced significant levels of antibody as detected by ELISA. On the other hand, all of the transfections using bicistronic UCOE generated viable parental transfectomas that produced significant levels of antibody.

One explanation could be that when monocistronic UCOE vectors were employed, two separate vectors were transfected at the same time, one coding for the light chain (pAS-hVL-hCL) of the antibody and one for the heavy chain (pAS-hVH-hCH). Thus, cells could be predominantly transfected with either pAS-hVL-hCL (which would produce only the light chain that is secreted) or pAS-hVH-hCH (which would produce only the heavy chain that is not secreted) or with both vectors (which would produce the whole antibody that is secreted). Since both vectors contain the same (puromycin) resistance gene all cells successfully transfected with any one of or both vectors would be viable after selection with puromycin. This would give rise to viable cells that produced light chains, heavy chains or whole antibody. As the capture antibody used for detection of secreted H1C2 mAb in the medium was directed towards the Fc region (whole antibody), results would therefore be positive for populations of cells that predominantly produced the whole antibody and negative for those producing predominantly the light chain or heavy chain (Mateo et al. 1997). Ideally, different selection antibiotics should be used for each of the monocistronic vectors during co-transfection.

After selection and cloning the production rate of antibodies in a substantial number of clones fell, more in NS0 transfectomas and less in CHO transfectomas. This fall in production in NS0 transfectomas could be due to the fact that large genomic re-arrangements had occurred during amplification. This instability of a transfectoma may involve the silencing of the exogenous gene due to modifications such as methylation of CpG DNA sequences (Zhang et al. 2010), histone deacetylation and chromatin condensation (Kim et al. 2011) thus resulting in loss of high-producing clones. This phenomenon may have occurred more frequently in NS0 cells transfected with pFUSE vectors than in CHO cells transfected with UCOE vectors.

However, the reason why H1C2 mAb could not be expressed in NS0 cells using UCOE vectors or in CHO cells using pFUSE vectors could not be determined and warrants further investigation. To our knowledge, H1C2 mAbs have previously been only produced using NS0 cells and this will be the first time this antibody is produced in CHO cells which are preferred over NS0 cells (Cacciatore et al. 2010). We feel that the procedure of transfecting CHO with UCOE vector is more advantageous in the industrial production of these humanized H1C2 mAb and may serve as a general template for production of other mAbs.

## Conclusion

From the experiments described in this article several conclusions may be drawn. First, the use of synthetic DNA coding the variable regions of H1C2 mAb provides a convenient and fast route to develop H1C2 mAbs which remain functional. Second, irrespective of the vector DNA size, the linearization of vectors increases the transfection efficiency; however the restriction enzyme must be chosen with careful consideration. Third, pFUSE expression vectors work well in NS0 cells and UCOE expression vectors work well in CHO cells. Fourth, it is advisable to use two different selection antibiotics for co-transfection performed using two monocistronic vectors, in order to minimize the probability of obtaining transfectomas expressing only the light chain of the antibody. Finally, transfection of CHO cells with UCOE vectors was more effective in generating a greater number of high-producing and stable clones, making it much easier and faster to find clones with the high productivity and stability required for manufacturing. In addition, this is the first time H1C2 mAb has been expressed in CHO cells which is the preferred cell line in manufacturing. Therefore these H1C2 mAbs could now be conveniently produced at large-scale at a reduced cost.

## Abbreviations

Cat. no.: Catalog number
CHO: Chinese hamster ovary
CHO-UCOE-B: CHO cells with bicistronic UCOE expression vector
CHO-UCOE-M: CHO cells with monocistronic UCOE expression vectors
Fc: Fragment-crystallizable
FITC: Fluorescein isothiocyanate
FU: Fluorescence units
H1C2: Humanized anti-C2 mAb developed using deimmunization method
mAb: Monoclonal antibody
NS0-pFUSE-M: NS0 cells with monocistronic pFUSE expression vectors
UCOE: Ubiquitous chromatin opening element
VH: Variable region of antibody heavy chain
VL: Variable region of antibody light chain
